# 2-Azido­methyl-3-methyl-1-phenyl­sulfonyl-1*H*-indole

**DOI:** 10.1107/S1600536811030601

**Published:** 2011-08-06

**Authors:** S. Karthikeyan, K. Sethusankar, Ganesan Gobi Rajeswaran, Arasambattu K. Mohanakrishnan

**Affiliations:** a Department of Physics, RKM Vivekananda College (Autonomous), Chennai 600 004, India; b Department of Organic Chemistry, University of Madras, Maraimalai Campus, Chennai 600 025, India

## Abstract

In the title compound, C_16_H_14_N_4_O_2_S, the plane of the indole ring is twisted by 70.4 (2)° with respect to the plane of the azidomethyl­ substituent. As a result of the electron-withdrawing character of the phenyl­sulfonyl groups, the N—C bond lengths are slightly longer than the anti­cipated value of approximately 1.355 Å for an N atom with a planar configuration. The indole ring is essentially planar, with a maximum deviation of 0.0296 Å. The azide group is almost linear, the N—N—N angle being 171.4 (3)°. The methyl group on the azide-substituted C atom is in a flagpole position. The phenyl ring of the sulfonyl substituent makes a dihedral angle of 87.07 (10)° with the best plane of the indole moiety. The crystal packing is stabilized by inter­molecular C—H⋯O inter­actions, which link the mol­ecules into infinite chains running parallel to the *b* axis. The crystal packing is further stabilized by C—H⋯π inter­actions.

## Related literature

For the biological activity of compounds containing an indole ring system, sulfur and azides, see: Williams *et al.* (1993 ▶); Amblard *et al.* (2009 ▶); De-Benedetti *et al.* (1985 ▶). For related structures, see: Fernandes *et al.* (2005 ▶). For comparison of mol­ecular dimensions, see: Bassindale (1984 ▶); Allen *et al.* (1987 ▶).
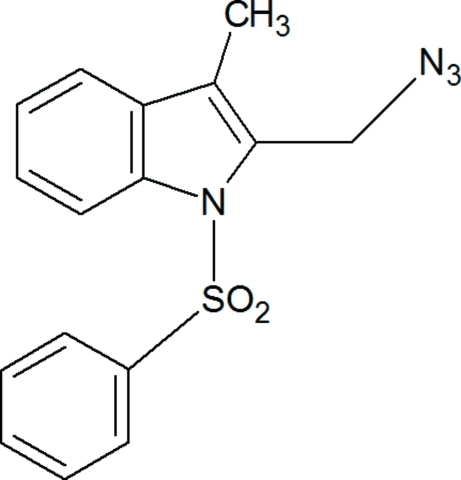


## Experimental

### 

#### Crystal data

C_16_H_14_N_4_O_2_S
*M* *_r_* = 326.38
Orthorhombic, 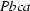

*a* = 11.0337 (4) Å
*b* = 12.1424 (4) Å
*c* = 23.2234 (9) Å
*V* = 3111.37 (19) Å^3^
*Z* = 8
Mo *K*α radiation
μ = 0.22 mm^−1^
*T* = 295 K
0.30 × 0.25 × 0.25 mm

#### Data collection

Bruker Kappa APEXII CCD diffractometer
37510 measured reflections
4231 independent reflections
2776 reflections with *I* > 2σ(*I*)
*R*_int_ = 0.049

#### Refinement

*R*[*F* ^2^ > 2σ(*F* ^2^)] = 0.047
*wR*(*F* ^2^) = 0.166
*S* = 0.99
4231 reflections
209 parameters
H-atom parameters constrained
Δρ_max_ = 0.37 e Å^−3^
Δρ_min_ = −0.35 e Å^−3^

### 

Data collection: *APEX2* (Bruker, 2004 ▶); cell refinement: *SAINT* (Bruker, 2004 ▶); data reduction: *SAINT*; program(s) used to solve structure: *SHELXS97* (Sheldrick, 2008 ▶); program(s) used to refine structure: *SHELXL97* (Sheldrick, 2008 ▶); molecular graphics: *ORTEP-3* (Farrugia, 1997 ▶); software used to prepare material for publication: *SHELXL97* and *PLATON* (Spek, 2009 ▶).

## Supplementary Material

Crystal structure: contains datablock(s) global, I. DOI: 10.1107/S1600536811030601/rk2287sup1.cif

Structure factors: contains datablock(s) I. DOI: 10.1107/S1600536811030601/rk2287Isup2.hkl

Supplementary material file. DOI: 10.1107/S1600536811030601/rk2287Isup3.cml

Additional supplementary materials:  crystallographic information; 3D view; checkCIF report

## Figures and Tables

**Table 1:** Hydrogen-bond geometry (Å, °) *Cg*3 is the centroid of the C11–C16 ring.

*D*—H⋯*A*	*D*—H	H⋯*A*	*D*⋯*A*	*D*—H⋯*A*
C14—H14⋯O1^i^	0.93	2.46	3.322 (3)	154
C5—H5⋯*Cg*3^ii^	0.93	2.89	3.714 (3)	148

Symmetry codes: (i) 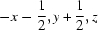; (ii) 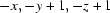.

**Fig. 1.**
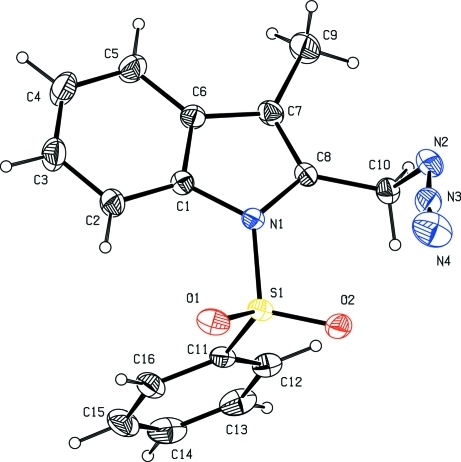
The molecular structure of the title compound with the atom numbering scheme. Displacement ellipsoids are drawn at 30% probability level. H atoms are present as a small spheres of arbitary radius.

**Fig. 2.**
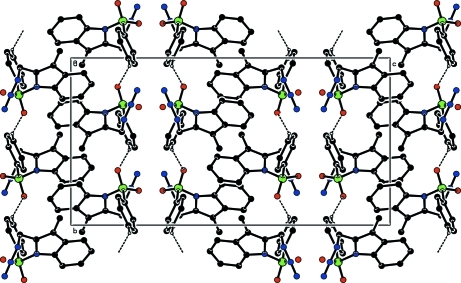
The packing arrangement of the title compound viewed down a axis. The dashed lines indicate C—H···O intermolecular interactions, which is running parallel to b axis. Initial symmetry code: (i) -1/2-x, 1/2+y, z.

## supplementary crystallographic information

### Comment

The phenylsulfonyl indole compounds inhibit the HIV–1 RT enzyme *in vitro* and HTLVIIIb viral spread in MT–4 human T–lymphoid cells (Williams, *et al.*, 1993). The Cu(I)–catalyzed 1,3–dipolar cycloaddition reaction between alkynes and azides has been suitable for the synthesis of a large number of modified nucleosides, nucleotides and oligonucleotides with a broad range of applications (Amblard *et al.*, 2009). A lot of sulfur containing compounds, exhibit insecticidal, germicidal, antimicrobial and antibacterial activities (De-Benedetti *et al.*, 1985).

In the title compound C_16_H_14_N_4_O_2_S, the molecular conformation (Fig. 1) is preferred with the plane of indole ring twisted by 70.4 (2)° with respect to the plane of the azido group bound to the methyl substituent. The indole ring is essentially planar with a maximum deviation 0.0296 (17)Å for the atom N1. The bond angle around N3, in the chain of atom N2–N3–N4, is 171.4 (3)° and thus the azidomethyl side chain is almost linear. The methyl group on the azide substituted C atom is in a flag pole position.

The phenyl ring of the sulfonyl substituent makes a dihedral angle of 87.07 (10)° with the indole moiety. The deviation of atoms S1 and C10 from the indole mean plane is 0.453 (5)Å and -0.0618 (24)Å, respectively. As a result of electron–withdrawing character of the phenylsulfonyl group, the bond lengths N1—C8 = 1.432 (2)Å and N1—C1 = 1.416 (2)Å in the molecule are longer than the mean value of 1.355 (14)Å (Allen *et al.*, 1987). Due to Thorpe–Ingold effect (Bassindale, 1984), bond angles around atom S1 show significant deviations from the ideal tetrahedral value, with significant deviations, widening of angle O1═S1═O2 = 119.71 (10)° and narrowing of angle N1—S1—C11 = 105.36 (8)°. The title molecule exhibits structural similarities with the already reported related structure (Fernandes *et al.*, 2005).

In crystal packing, the molecule is stabilized by intermolecular C—H···O interactions which link the molecules into infinite chains running parallel to *b* axis. The crystal packing is further stabilized by C—H···π interaction, where *Cg*3 is centroid of C11–C16. The symmetry codes: (i) -1/2-*x*, 1/2+*y*, *z*; (ii) -*x*, 1-*y*, 1-*z*. The packing view of the title compound is shown in the Fig. 2.

### Experimental

To a solution of 2–(bromomethyl)–3–methyl–1–phenylsulfonyl–indole (1 mmol) in *DMF* (3 ml) was added sodium azide (2 mmol) and stirred for 2 h at room temperature. After consumption of the 2–(bromomethyl)–3–methyl–1–phenylsulfonyl–indole (monitored by *TLC*), reaction mass was poured into ice water (20 ml). The solid obtained was filtered and dried (CaSO_4_). Then the crude product was recrystalized with *Me*OH (5 ml) afforded the 2–(azidomethyl)–3–methyl–1–phenylsulfonyl–indole as a colourless solid. Yield: 0.28 g (92%).

### Refinement

All the hydrogen atoms in the molecule were placed geometrically and allowed to ride on their parent atoms with C—H distance in the range 0.93Å to 0.97Å and with *U*_iso_(H) = 1.5*U*_eq_(C) for CH_3_ group and *U*_iso_(H) = 1.2*U*_eq_(C) for all the other groups.

### Crystal data

**Table:**

C_16_H_14_N_4_O_2_S	*F*(000) = 1360
*M**_r_* = 326.38	*D*_x_ = 1.393 Mg m^−^^3^
Orthorhombic, *P**b**c**a*	Mo *K*α radiation, λ = 0.71073 Å
Hall symbol: -P 2ac 2ab	Cell parameters from 4231 reflections
*a* = 11.0337 (4) Å	θ = 1.0–29.3°
*b* = 12.1424 (4) Å	µ = 0.22 mm^−^^1^
*c* = 23.2234 (9) Å	*T* = 295 K
*V* = 3111.37 (19) Å^3^	Block, colourless
*Z* = 8	0.30 × 0.25 × 0.25 mm

### Data collection

**Table:**

Bruker Kappa APEXII CCD diffractometer	2776 reflections with *I* > 2σ(*I*)
Radiation source: fine–focus sealed tube	*R*_int_ = 0.049
graphite	θ_max_ = 29.3°, θ_min_ = 2.6°
ω scans	*h* = −15→13
37510 measured reflections	*k* = −16→12
4231 independent reflections	*l* = −31→31

### Refinement

**Table:**

Refinement on *F*^2^	Primary atom site location: structure-invariant direct methods
Least-squares matrix: full	Secondary atom site location: difference Fourier map
*R*[*F*^2^ > 2σ(*F*^2^)] = 0.047	Hydrogen site location: inferred from neighbouring sites
*wR*(*F*^2^) = 0.166	H-atom parameters constrained
*S* = 0.99	*w* = 1/[σ^2^(*F*_o_^2^) + (0.0987*P*)^2^ + 0.4179*P*] where *P* = (*F*_o_^2^ + 2*F*_c_^2^)/3
4231 reflections	(Δ/σ)_max_ < 0.001
209 parameters	Δρ_max_ = 0.37 e Å^−^^3^
0 restraints	Δρ_min_ = −0.35 e Å^−^^3^

### Special details

**Table:**

Geometry. All s.u.'s (except the s.u. in the dihedral angle between two l.s. planes) are estimated using the full covariance matrix. The cell s.u.'s are taken into account individually in the estimation of s.u.'s in distances, angles and torsion angles; correlations between s.u.'s in cell parameters are only used when they are defined by crystal symmetry. An approximate (isotropic) treatment of cell s.u.'s is used for estimating s.u.'s involving l.s. planes.
Refinement. Refinement of *F*^2^ against ALL reflections. The weighted *R*–factor *wR* and goodness of fit *S* are based on *F*^2^, conventional *R*–factors *R* are based on *F*, with *F* set to zero for negative *F*^2^. The threshold expression of *F*^2^ > σ(*F*^2^) is used only for calculating *R*–factors(gt) *etc*. and is not relevant to the choice of reflections for refinement. *R*–factors based on *F*^2^ are statistically about twice as large as those based on *F*, and *R*–factors based on ALL data will be even larger.

### Fractional atomic coordinates and isotropic or equivalent isotropic displacement parameters (Å^2^)

**Table:**

	*x*	*y*	*z*	*U*_iso_*/*U*_eq_	
C1	0.07440 (17)	0.32220 (15)	0.44968 (8)	0.0419 (4)	
C2	−0.0143 (2)	0.25607 (19)	0.47362 (10)	0.0566 (5)	
H2	−0.0569	0.2052	0.4516	0.068*	
C3	−0.0368 (3)	0.2688 (2)	0.53153 (12)	0.0719 (7)	
H3	−0.0958	0.2253	0.5488	0.086*	
C4	0.0252 (3)	0.3435 (3)	0.56443 (11)	0.0807 (8)	
H4	0.0075	0.3495	0.6034	0.097*	
C5	0.1128 (2)	0.4095 (2)	0.54104 (10)	0.0690 (7)	
H5	0.1548	0.4600	0.5636	0.083*	
C6	0.13730 (18)	0.39903 (16)	0.48237 (9)	0.0478 (5)	
C7	0.22068 (19)	0.45506 (16)	0.44500 (9)	0.0513 (5)	
C8	0.20845 (16)	0.41425 (15)	0.39159 (8)	0.0424 (4)	
C9	0.3091 (3)	0.5412 (2)	0.46516 (13)	0.0844 (9)	
H9A	0.3390	0.5816	0.4326	0.127*	
H9B	0.3756	0.5062	0.4846	0.127*	
H9C	0.2691	0.5908	0.4912	0.127*	
C10	0.2829 (2)	0.4427 (2)	0.34045 (9)	0.0529 (5)	
H10A	0.3110	0.5181	0.3441	0.064*	
H10B	0.2328	0.4382	0.3062	0.064*	
C11	−0.08441 (17)	0.36552 (16)	0.33108 (8)	0.0433 (4)	
C12	−0.0704 (2)	0.46737 (18)	0.30491 (9)	0.0547 (5)	
H12	0.0048	0.4893	0.2910	0.066*	
C13	−0.1695 (3)	0.5355 (2)	0.29992 (11)	0.0711 (7)	
H13	−0.1615	0.6043	0.2828	0.085*	
C14	−0.2791 (3)	0.5020 (3)	0.32010 (13)	0.0801 (9)	
H14	−0.3457	0.5484	0.3164	0.096*	
C15	−0.2932 (2)	0.4012 (3)	0.34580 (12)	0.0780 (8)	
H15	−0.3690	0.3797	0.3592	0.094*	
C16	−0.19488 (19)	0.3311 (2)	0.35192 (11)	0.0609 (6)	
H16	−0.2033	0.2628	0.3696	0.073*	
N1	0.12022 (14)	0.32780 (13)	0.39281 (6)	0.0405 (3)	
N2	0.38932 (18)	0.3686 (2)	0.33357 (9)	0.0696 (6)	
N3	0.37305 (17)	0.2784 (2)	0.31151 (9)	0.0632 (5)	
N4	0.3730 (2)	0.1938 (2)	0.29245 (13)	0.0975 (8)	
O1	0.00404 (15)	0.17244 (11)	0.35322 (7)	0.0599 (4)	
O2	0.11681 (14)	0.29357 (13)	0.28769 (6)	0.0590 (4)	
S1	0.04241 (4)	0.27961 (4)	0.33704 (2)	0.04300 (17)	

### Atomic displacement parameters (Å^2^)

**Table:**

	*U*^11^	*U*^22^	*U*^33^	*U*^12^	*U*^13^	*U*^23^
C1	0.0427 (10)	0.0382 (9)	0.0449 (10)	−0.0001 (8)	0.0005 (8)	0.0030 (7)
C2	0.0576 (13)	0.0529 (11)	0.0593 (13)	−0.0137 (10)	0.0052 (10)	0.0041 (10)
C3	0.0766 (17)	0.0759 (17)	0.0633 (15)	−0.0133 (13)	0.0191 (13)	0.0112 (12)
C4	0.103 (2)	0.0881 (19)	0.0509 (14)	−0.0151 (17)	0.0197 (14)	−0.0017 (12)
C5	0.0830 (17)	0.0747 (15)	0.0494 (12)	−0.0168 (14)	0.0032 (12)	−0.0107 (11)
C6	0.0500 (11)	0.0466 (11)	0.0468 (10)	−0.0046 (9)	−0.0014 (8)	−0.0035 (8)
C7	0.0491 (11)	0.0467 (11)	0.0580 (12)	−0.0103 (9)	−0.0008 (9)	−0.0061 (9)
C8	0.0363 (9)	0.0417 (9)	0.0490 (10)	−0.0026 (7)	−0.0004 (7)	0.0029 (7)
C9	0.0866 (19)	0.0825 (18)	0.0841 (19)	−0.0432 (16)	0.0045 (15)	−0.0183 (14)
C10	0.0471 (11)	0.0592 (12)	0.0524 (12)	−0.0067 (10)	0.0018 (9)	0.0077 (9)
C11	0.0385 (9)	0.0463 (10)	0.0452 (10)	0.0038 (8)	−0.0076 (8)	−0.0100 (8)
C12	0.0605 (13)	0.0535 (12)	0.0501 (11)	0.0067 (10)	−0.0110 (10)	−0.0028 (9)
C13	0.089 (2)	0.0627 (14)	0.0613 (14)	0.0285 (14)	−0.0205 (13)	−0.0114 (11)
C14	0.0729 (19)	0.089 (2)	0.0786 (18)	0.0410 (16)	−0.0250 (15)	−0.0325 (15)
C15	0.0385 (12)	0.107 (2)	0.0881 (19)	0.0117 (13)	−0.0040 (11)	−0.0294 (16)
C16	0.0414 (11)	0.0694 (15)	0.0719 (14)	0.0003 (10)	−0.0048 (10)	−0.0123 (11)
N1	0.0381 (8)	0.0434 (8)	0.0400 (8)	−0.0047 (6)	−0.0029 (6)	0.0015 (6)
N2	0.0389 (10)	0.0972 (17)	0.0728 (14)	−0.0009 (11)	0.0019 (9)	−0.0075 (11)
N3	0.0461 (10)	0.0842 (17)	0.0594 (12)	0.0081 (11)	0.0006 (9)	0.0063 (11)
N4	0.0768 (17)	0.0868 (18)	0.129 (2)	0.0254 (14)	−0.0104 (16)	−0.0159 (17)
O1	0.0639 (10)	0.0375 (8)	0.0783 (10)	−0.0019 (7)	−0.0153 (8)	−0.0086 (7)
O2	0.0488 (8)	0.0817 (11)	0.0463 (8)	0.0069 (7)	0.0009 (6)	−0.0149 (7)
S1	0.0380 (3)	0.0433 (3)	0.0478 (3)	0.00322 (19)	−0.00599 (19)	−0.00836 (18)

### Geometric parameters (Å, °)

**Table:**

C1—C2	1.383 (3)	C10—H10A	0.9700
C1—C6	1.389 (3)	C10—H10B	0.9700
C1—N1	1.416 (2)	C11—C16	1.377 (3)
C2—C3	1.376 (4)	C11—C12	1.387 (3)
C2—H2	0.9300	C11—S1	1.7509 (19)
C3—C4	1.369 (4)	C12—C13	1.376 (3)
C3—H3	0.9300	C12—H12	0.9300
C4—C5	1.368 (4)	C13—C14	1.359 (4)
C4—H4	0.9300	C13—H13	0.9300
C5—C6	1.395 (3)	C14—C15	1.371 (4)
C5—H5	0.9300	C14—H14	0.9300
C6—C7	1.436 (3)	C15—C16	1.386 (3)
C7—C8	1.342 (3)	C15—H15	0.9300
C7—C9	1.505 (3)	C16—H16	0.9300
C8—N1	1.432 (2)	N1—S1	1.6604 (15)
C8—C10	1.485 (3)	N2—N3	1.222 (3)
C9—H9A	0.9600	N3—N4	1.119 (3)
C9—H9B	0.9600	O1—S1	1.4190 (16)
C9—H9C	0.9600	O2—S1	1.4200 (15)
C10—N2	1.488 (3)		
			
C2—C1—C6	121.61 (19)	C8—C10—H10B	109.1
C2—C1—N1	130.98 (18)	N2—C10—H10B	109.1
C6—C1—N1	107.41 (16)	H10A—C10—H10B	107.9
C3—C2—C1	117.1 (2)	C16—C11—C12	121.6 (2)
C3—C2—H2	121.5	C16—C11—S1	119.92 (17)
C1—C2—H2	121.5	C12—C11—S1	118.48 (16)
C4—C3—C2	122.0 (2)	C13—C12—C11	119.0 (2)
C4—C3—H3	119.0	C13—C12—H12	120.5
C2—C3—H3	119.0	C11—C12—H12	120.5
C5—C4—C3	121.3 (2)	C14—C13—C12	119.9 (3)
C5—C4—H4	119.4	C14—C13—H13	120.0
C3—C4—H4	119.4	C12—C13—H13	120.0
C4—C5—C6	118.1 (2)	C13—C14—C15	121.2 (2)
C4—C5—H5	120.9	C13—C14—H14	119.4
C6—C5—H5	120.9	C15—C14—H14	119.4
C1—C6—C5	119.90 (19)	C14—C15—C16	120.3 (3)
C1—C6—C7	107.95 (17)	C14—C15—H15	119.9
C5—C6—C7	132.2 (2)	C16—C15—H15	119.9
C8—C7—C6	108.61 (17)	C11—C16—C15	118.0 (3)
C8—C7—C9	127.5 (2)	C11—C16—H16	121.0
C6—C7—C9	123.8 (2)	C15—C16—H16	121.0
C7—C8—N1	108.70 (16)	C1—N1—C8	107.24 (14)
C7—C8—C10	126.69 (18)	C1—N1—S1	121.74 (13)
N1—C8—C10	124.23 (17)	C8—N1—S1	126.47 (12)
C7—C9—H9A	109.5	N3—N2—C10	118.10 (19)
C7—C9—H9B	109.5	N4—N3—N2	171.4 (3)
H9A—C9—H9B	109.5	O1—S1—O2	119.71 (10)
C7—C9—H9C	109.5	O1—S1—N1	105.72 (9)
H9A—C9—H9C	109.5	O2—S1—N1	106.78 (9)
H9B—C9—H9C	109.5	O1—S1—C11	109.20 (10)
C8—C10—N2	112.42 (17)	O2—S1—C11	109.09 (9)
C8—C10—H10A	109.1	N1—S1—C11	105.36 (8)
N2—C10—H10A	109.1		
			
C6—C1—C2—C3	−0.7 (3)	C13—C14—C15—C16	0.3 (4)
N1—C1—C2—C3	178.5 (2)	C12—C11—C16—C15	0.3 (3)
C1—C2—C3—C4	0.1 (4)	S1—C11—C16—C15	−179.36 (17)
C2—C3—C4—C5	0.1 (5)	C14—C15—C16—C11	−0.6 (4)
C3—C4—C5—C6	0.2 (4)	C2—C1—N1—C8	177.9 (2)
C2—C1—C6—C5	1.0 (3)	C6—C1—N1—C8	−2.8 (2)
N1—C1—C6—C5	−178.3 (2)	C2—C1—N1—S1	20.4 (3)
C2—C1—C6—C7	−179.01 (19)	C6—C1—N1—S1	−160.34 (14)
N1—C1—C6—C7	1.6 (2)	C7—C8—N1—C1	3.1 (2)
C4—C5—C6—C1	−0.8 (4)	C10—C8—N1—C1	176.49 (18)
C4—C5—C6—C7	179.3 (2)	C7—C8—N1—S1	159.22 (15)
C1—C6—C7—C8	0.3 (2)	C10—C8—N1—S1	−27.4 (3)
C5—C6—C7—C8	−179.7 (2)	C8—C10—N2—N3	80.5 (3)
C1—C6—C7—C9	−177.3 (2)	C1—N1—S1—O1	−48.72 (17)
C5—C6—C7—C9	2.7 (4)	C8—N1—S1—O1	158.31 (15)
C6—C7—C8—N1	−2.1 (2)	C1—N1—S1—O2	−177.23 (14)
C9—C7—C8—N1	175.4 (2)	C8—N1—S1—O2	29.80 (18)
C6—C7—C8—C10	−175.29 (19)	C1—N1—S1—C11	66.85 (16)
C9—C7—C8—C10	2.2 (4)	C8—N1—S1—C11	−86.12 (17)
C7—C8—C10—N2	91.2 (3)	C16—C11—S1—O1	12.21 (19)
N1—C8—C10—N2	−81.0 (2)	C12—C11—S1—O1	−167.48 (15)
C16—C11—C12—C13	0.2 (3)	C16—C11—S1—O2	144.74 (17)
S1—C11—C12—C13	179.90 (16)	C12—C11—S1—O2	−34.96 (17)
C11—C12—C13—C14	−0.5 (3)	C16—C11—S1—N1	−100.94 (17)
C12—C13—C14—C15	0.3 (4)	C12—C11—S1—N1	79.36 (16)

### Hydrogen-bond geometry (Å, °)

**Table:**

Cg3 is the centroid of the C11–C16 ring.

**Table:**

*D*—H···*A*	*D*—H	H···*A*	*D*···*A*	*D*—H···*A*
C14—H14···O1^i^	0.93	2.46	3.322 (3)	154.
C5—H5···Cg3^ii^	0.93	2.89	3.714 (3)	148

Symmetry codes: (i) −*x*−1/2, *y*+1/2, *z*; (ii) −*x*, −*y*+1, −*z*+1.
